# Imbalanced Multi-Modal Multi-Label Learning for Subcellular Localization Prediction of Human Proteins with Both Single and Multiple Sites

**DOI:** 10.1371/journal.pone.0037155

**Published:** 2012-06-08

**Authors:** Jianjun He, Hong Gu, Wenqi Liu

**Affiliations:** School of Control Science and Engineering, Dalian University of Technology, Dalian, Liaoning, China; King's College London, United Kingdom

## Abstract

It is well known that an important step toward understanding the functions of a protein is to determine its subcellular location. Although numerous prediction algorithms have been developed, most of them typically focused on the proteins with only one location. In recent years, researchers have begun to pay attention to the subcellular localization prediction of the proteins with multiple sites. However, almost all the existing approaches have failed to take into account the correlations among the locations caused by the proteins with multiple sites, which may be the important information for improving the prediction accuracy of the proteins with multiple sites. In this paper, a new algorithm which can effectively exploit the correlations among the locations is proposed by using Gaussian process model. Besides, the algorithm also can realize optimal linear combination of various feature extraction technologies and could be robust to the imbalanced data set. Experimental results on a human protein data set show that the proposed algorithm is valid and can achieve better performance than the existing approaches.

## Introduction

Over the past years, the research on determining the subcellular locations of proteins has attracted more attention from academia due to its important roles in understanding protein functions, identifying drug targets, annotating genomes and so on. The approaches for determining the subcellular locations of proteins can be divided into two categories: experimental and computational methods [1]. Experimental methods such as cell fractionation, electron microscopy and fluorescence microscopy usually are time consuming, expensive and laborious [2]. These limitations have made the experimental methods unable to cope with the situation that a large number of protein sequences continue to emerge from the genome sequencing projects, and have encouraged the ongoing efforts to develop computational methods. It is well known that the information on the final subcellular location of a protein is basically encoded as a part of its amino acid sequence and such a sequence is thought to be recognized by a specific receptor protein as a protein sorting signal. Thus, it would be possible, at least in principle, for us to predict the subcellular location of a protein from its amino acid sequence by using computational methods [3]. In addition, many studies in other related areas have indicated that sequence-based prediction approaches, such as those for predicting drug-target interaction networks [4], predicting transcriptional activity of multiple site p53 mutants [5], prediction of body fluids [6], predicting protein metabolic stability [7], predicting antimicrobial peptides [8], identifying DNA binding proteins [9], identifying regulatory pathways [10], predicting signal peptides [11], predicting HIV cleavage sites in proteins [12], [13], predicting the network of substrate-enzyme-product triads [14], predicting protein pathway networks [15], predicting proteases and their types [16], and predicting membrane proteins and their types [17], can generate many useful data for which it would be time-consuming and costly to obtain by experiments alone, and can timely provide very useful insights for both basic research and application by being combined with the information derived from the structural bioinformatics tools (see, e.g., [18]). In view of this, computationally predicting the subcellular locations of proteins from their amino acid sequences may become a useful complement to the experimental methods.

Since the pioneering efforts were provided [19], [20], a number of sequence-based computational methods had been developed for predicting the subcellular locations of proteins. For example, based on N-terminal sequence information only, a neural network-based tool called TargetP was developed in [21] for large-scale subcellular localization prediction. Support vector machine (SVM) was introduced to predict the subcellular locations of proteins from their amino acid composition [22] and functional domain composition [23], respectively. In [24], [25], the subcellular localization prediction problem of apoptosis proteins was studied. In order to avoid losing the sequence order information, Chou [26] proposed a concept of pseudo amino acid composition (PseAA composition) to represent the protein samples. Soon afterwards, many different prediction methods were proposed based on PseAA composition [27]–[34]. Text mining approach was used to improve the prediction results of protein subcellular localization by Lu et al. [35] for both prokaryote and eukaryote. MultiLoc, a SVM-based approach, was proposed in [36] through integrating N-terminal targeting sequences, amino acid composition and protein sequence motifs. A package of web servers named Cell-PLoc was developed by Chou and Shen [37] for predicting the subcellular locations of proteins in various organisms. A wider view of some other published protein subcellular localization prediction methods may be found in [2], [3].

**Figure 1:**
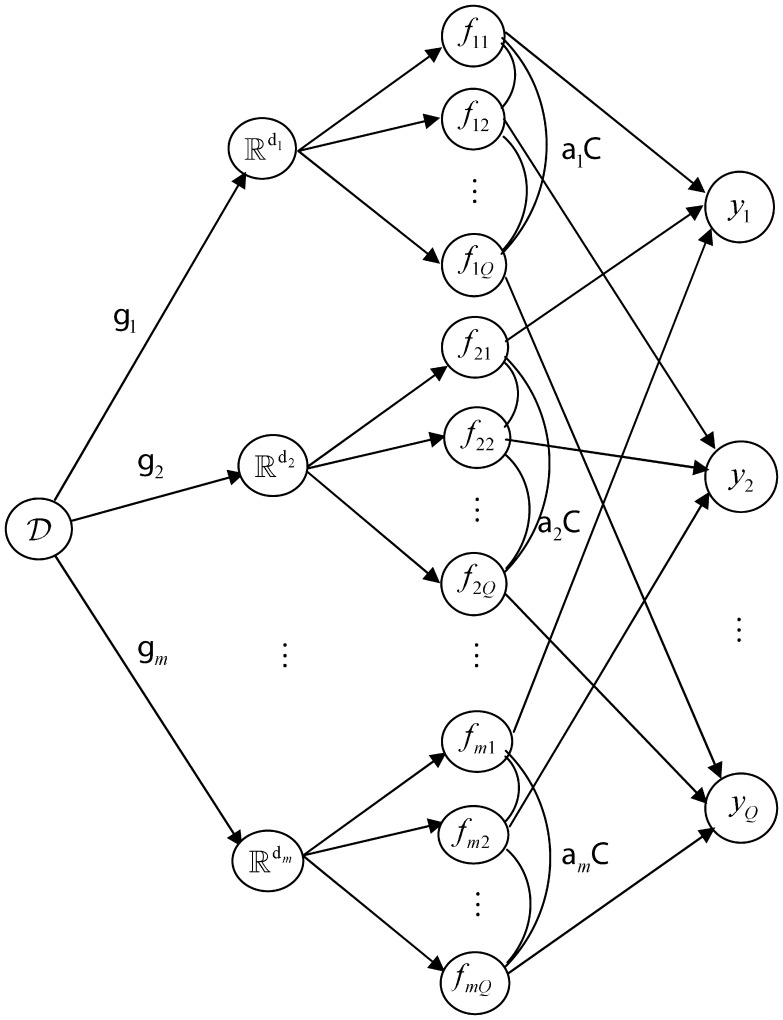
Graphical model for IMMMLGP.

As mentioned above, through the continuing efforts of researchers, many computational methods which can achieve superior performance have been developed. However, all these studies [1]–[3], [19]–[39], except for [2] and [37], focused only on mono-locational proteins, i.e., they assume that each protein exists in only one cellular compartment. This is not always the case. In fact, recent evidences [40], [41] indicate that a mass of proteins have multiple sites in the cell. For addressing this problem, Scott et al. [42] established a Bayesian network predictor based on the combination of InterPro motifs and specific membrane domains in human proteins. By hybridizing three feature extraction techniques including gene ontology, functional domain and pseudo amino acid composition, Chou and Cai [43] developed a nearest neighbor algorithm for predicting the subcellular locations of proteins with multiple sites in budding yeast. In 2007, based on a feature representation frame of hybridizing gene ontology and amphiphilic pseudo amino acid composition and an ensemble k-nearest neighbor classifier, two algorithms called Euk-mPLoc [44] and Hum-mPLoc [45] were developed by Chou and Shen to deal with the eukaryotic and human proteins with both single and multiple sites, respectively. Later, they presented an improved feature representation frame by hybridizing the gene ontology, functional domain, and sequential evolutionary information, and several new approaches such as Euk-mPLoc 2.0 [46], Hum-mPLoc 2.0 [47], Plant-mPLoc [48] and Virus-mPLoc [49] were proposed. Lee et al. [50] developed a PLPD algorithm by using a density-induced support vector data description (D-SVDD) approach. In [51], Briesemeister et al. presented an algorithm named YLoc by using the simple naive Bayes classifier. Lin et al. [52] proposed a knowledge based approach by using the local sequence similarity. Recently, four new approaches called iLoc-Euk [53], iLoc-Gneg [54], iLoc-Plant [55] and iLoc-Virus [56] were proposed based on a multi-label classifier to predict the subcellular locations of eukaryotic, Gram-negative bacterial, plant, and virus proteins, respectively. In [57], Wu et al. presented a multi-layer classifier to predict the subcellular locations of Gram-positive bacterial proteins. In [58], a new predictor, called iLoc-Hum, was developed based on the accumulation-label scale for predicting the subcellular locations of human proteins.

**Table 1:** The experimental results (mean) on human protein data sets for investigating the usefulness of the correlations among the locations.

Evaluation metric		The proposed algorithm					
		The original data set			The new data set (40%)		
		Normal	Variation	The gap	Normal	Variation	The gap
The whole test set	Average precision 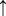	0.661	0.655	0.006	0.653	0.636	0.017
	Recall 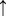	0.595	0.587	0.008	0.562	0.543	0.019
	F1-score 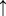	0.530	0.522	0.008	0.516	0.504	0.012
	Absolute true success rate 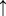	0.274	0.261	0.013	0.204	0.189	0.015
	Coverage 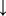	2.003	2.047	−0.044	2.630	2.711	−0.081
	Ranking loss 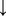	0.129	0.132	−0.003	0.143	0.148	−0.005
Samples withmultiple sites	Average precision 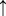	0.688	0.673	0.015	0.700	0.678	0.022
	Recall 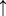	0.478	0.459	0.019	0.535	0.498	0.037
	F1-score 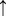	0.535	0.518	0.017	0.572	0.545	0.027
	Absolute true success rate 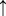	0.179	0.148	0.031	0.231	0.181	0.050
	Coverage 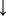	3.889	4.030	−0.141	3.825	3.954	−0.129
	Ranking loss 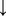	0.152	0.158	−0.006	0.148	0.155	−0.007

In order to deal with the protein with multiple sites, the common idea of the existing approaches is to train one or more single-label classifiers by transforming the original multi-label data into single-label ones and classify the query protein to the locations whose score outputted by the single-label classifiers satisfying some conditions. Three strategies were mainly used to transform the original multi-label data into single-label data. The first category such as Chou and Shen’s work [46], [49] is to take the protein with multiple sites as multiple proteins with single site; the second category [50] is to transform the original data set into multiple binary data sets, one for each location, and each binary data set includes all protein samples of the original data set, which are labeled positively if in the original data set they belong to the location corresponding to this binary data set and negatively otherwise; the third category [51] is to regard every possible combination of locations as a new class. However, the third strategy is infeasible in most cases because the number of classes will increase exponentially and the data in the new classes usually are sparse; the first and second one have limitations as well because they neglect the correlations among the locations caused by the protein with multiple sites. In fact, the correlations among the locations are the important information for improving the prediction accuracy. Taking the data set of eukaryotic proteins [46] as an example, it can be seen that almost all the proteins of cyanelle and hydrogenosome only have one site and about 30% proteins of cytoplasm also belong to nucleus. If a classifier can obtain these correlations from the training data set, it will think over the correctness of prediction result “a certain protein belongs to cyanelle and other locations simultaneously”, and will have to reconsider whether the location ‘nucleus’ is missed when a protein was located to cytoplasm only. Thus, the first research content of this paper is to improve the performance of the classifier by considering the correlations among the locations caused by the protein with multiple sites.

In addition, to improve the whole performance of protein subcellular localization prediction approaches, another important factor is to represent the proteins with an effective feature extraction technology. Although the proteins may contain all the information such that they can be transported to the due subcellular compartments exactly, to establish a quality feature extraction technology that can mine this information is still a challenging problem. However, with the efforts of researchers, various types of feature extraction technologies based on the different local information of proteins such as N-terminus, sequence motifs, amino acid composition, and gene ontology terms have been proposed. Thus, we can try to improve the prediction performance by incorporating multiple local feature information of proteins. In fact, researchers have already done some work in this aspect. However, in many cases, different types of feature information were included in one predictor based on the subjective understanding of researchers, and it is hardly to realize the optimal combination of them. Thus, the second research context of our work is to optimally combine multiple feature extraction technologies in the predictor.

Furthermore, the subcellular distribution of proteins is usually extremely imbalanced. For example, in the data set of eukaryotic proteins [46], the number of proteins in ‘cytoplasm’ is 2186, while the number of proteins in ‘Hydrogenosome’ is only 10. In this case, the common classifier will tend to be overwhelmed by the majority classes and ignore the minority ones. Thus, the third research context of our work is to address the imbalanced data problem.

In order to consider aforementioned three problems simultaneously, a new classifier is proposed in this paper by using Gaussian process model. The basic idea of the proposed algorithm is to define multiple latent functions on the feature spaces, then the correlations among the locations can be identified by the covariance matrix between these latent functions, the optimal linear combination of different feature extraction technologies can be realized by defining a likelihood function and the imbalance of data can be coped with by the weighting coefficient of the likelihood on each sample. Since it can deal with the problems possessing the following properties: (1) the distribution of data on different classes may be imbalanced, (2) the data are represented in multiple feature spaces, and (3) each datum may associate with multiple labels simultaneously, the machine learning framework described in this paper is named imbalanced multi-modal multi-label learning (IMMML). we also call the proposed algorithm imbalanced multi-modal multi-label Gaussian process (IMMMLGP).

According to a recent comprehensive review [59], to establish a really useful predictor for determining the subcellular locations of proteins based on their sequence information, we need to consider the following procedures: (i) construct or select a valid benchmark data set to train and test the predictor; (ii) formulate the protein samples with an effective mathematical expression that can truly reflect the intrinsic correlation with their subcellular locations; (iii) introduce or develop a powerful algorithm (or engine, classifier) to operate the prediction; (iv) properly perform cross-validation tests to objectively evaluate the anticipated accuracy of the predictor; (v) establish a user-friendly web-server for the predictor that is accessible to the public. Below, let us describe how to deal with these steps.

## Methods

Let *D* and 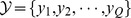 respectively denote the sets of proteins and the subcellular locations for a certain subcellular localization prediction problem, where *Q* is the number of subcellular locations. Let 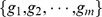 be the set of *m* feature extraction technologies used to extract the feature information of the proteins. Thus, each protein 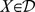 can be represented by 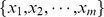, where, 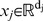 is the feature vector of *X* associated with 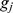, 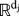 is the feature space corresponding to 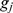, 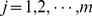. Suppose 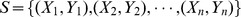 be a data set including *n* proteins with known sites, where, 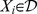 denotes the *i*th protein, 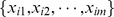 are the feature vectors of *X_i_* and 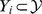 is the set of subcellular locations associated with *X_i_*, 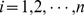. For notation’s convenience, *Y_i_* can be represented by a vector 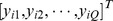, in which 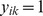 denotes that protein *X_i_* belongs to *y_k_*, otherwise 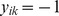. The goal of subcellular localization prediction of proteins with both single and multiple sites is to learn a function 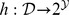 from *S* which can correctly predict the subcellular locations of a new protein 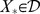. Being different with the traditional predictor for the proteins with single site, the output of *h* is a set of the locations.

Due to the desirable properties such as the natural Bayesian interpretation, explicit probabilistic formulation, and the ability to infer model parameters, Gaussian process model (GP) has received extensive attentions in recent years and become an important tool for many machine learning technologies. We will omit an introduction to it and refer the readers to the excellent books on this topic [60]. The main reason for using Gaussian process model but not other methods in this paper is that it can infer the correlations among the subcellular locations and the optimal combination coefficients of feature extraction technologies in a more convenient way.

To represent our uncertainty over subcellular locations for a protein, a better method is to output a probability for each subcellular location. As shown in Fig. 1, the main idea of IMMMLGP is to assume an unobservable latent function 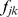 for every subcellular location *y_k_* on the feature space 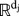, 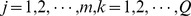, and then the probability that a protein *X* belongs to subcellular location *y_k_* can be obtained by the combination of latent functions 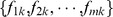 that assumed for *y_k_*. In IMMMLGP, the correlations among the subcellular locations can be identified by the covariance matrix of the latent functions; the optimal linear combination of different feature extraction technologies can be realized by defining a likelihood function and the combination coefficient of the *j*th feature extraction technology is just a parameter of the kernel function over feature space 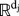; the imbalance of data can be coped with by giving a weighting coefficient to each sample in the joint likelihood. The details of IMMMLGP algorithm are shown as follows.

### Gaussian Process Prior

The basic idea behind Gaussian process model is to place a Gaussian process prior over the latent functions. In this paper, we place the Gaussian process priors with zero mean and the following covariance function over the latent functions 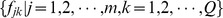,
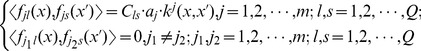
(1)where, 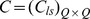 is a positive semi-definite matrix that specifies the correlations among the subcellular locations, so that the observation of one location can affect the prediction on another one. As will be seen from the Section “Joint Likelihood”, the main role of 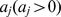 is the weighting coefficient of the *j*th feature extraction technology. *k^j^* is a covariance function over feature space 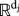. In this paper, the Gaussian kernel was used as the covariance function *k^j^*, i.e., 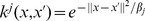. Since 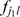 and 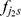 are the functions defined on different input spaces when 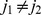, we can regard them as mutually independent functions.

We assume that all the parameters can be given except *C* and 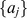. For notation’s convenience, let 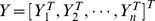, 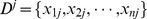, 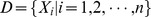, 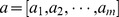, 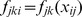, 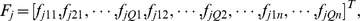 and 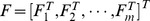, 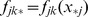, 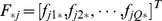, 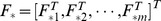, 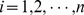, 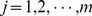, 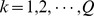, 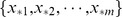 are the feature vectors of 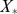.

According to (1), the joint distribution 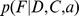 and 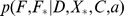 can be written as
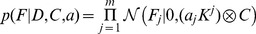
(2)and
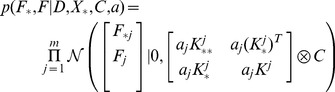
(3)respectively, where 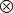 denotes the Kronecker product, the element of Kj is 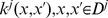, 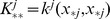, and 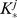 is a column vector and its ith element is 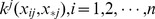. Thus, the conditional prior 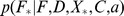 can be deduced analytically,
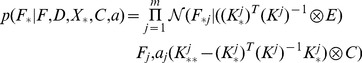
(4)where, E is an identity matrix.

### Joint Likelihood

Let 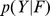 denote the joint likelihood, i.e., the joint probability of observing the class labels Y given the latent functions. Generally, the class labels can be regarded as independent variables given the latent functions. Thus, 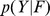 may be evaluated as a product of the likelihoods on individual observation, that is
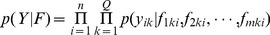
(5)Since the imbalance of data should be considered, we can set a weighting coefficient to the likelihood of each observation such that it can enhance the influence of minority classes on joint likelihood and reduce the influence of the majority classes, i.e.,
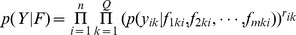
(6)A detailed explanation of why the likelihood (6) can deal with the imbalance of data and the details of determining 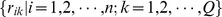 will be given in Appendix S1.

In this paper, we also would like to realize the optimal linear combination of various feature extraction technologies. It can be seen from (1) that the scale of 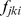 can be determined by the covariance function, this suggests that the linear combination 
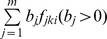
 of 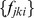 with covariance function 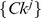 is equivalent to the sum 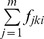 of 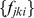 with covariance function 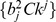. Thus, we can define likelihood 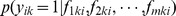 as
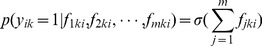
(7)then the optimal linear combination of various feature extraction technologies may be realized indirectly by choosing the weighting coefficients 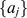 in (1). Here, 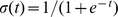 is the logistic function. As the values of 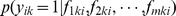 and 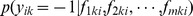 must sum to 1, thus likelihood 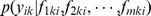 can be written as
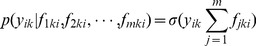
(8)


### Posterior Distribution

By using Bayes’s rule, the posterior distribution over *F* for given *C* and *a* becomes
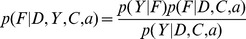
(9)where,
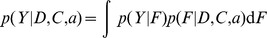
(10)is the marginal likelihood of the parameters C and a. It can be seen that the posterior distribution 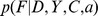 is a non-Gaussian distribution which can not be computed analytically. The same as the traditional GP classification models, Laplace’s method can be utilized to obtain a Gaussian approximation of 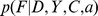, that is
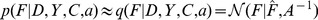
(11)where 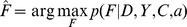 and 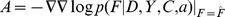 is the Hessian matrix of the negative log posterior at 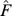. The details of solving 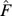 and A can be found in Appendix S1.

### Prediction

By using the approximation 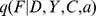 of posterior (9) and the conditional prior 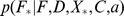 (4), the distribution of 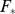 can be deduced analytically
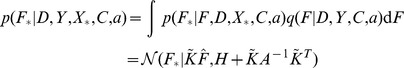
(12)where, 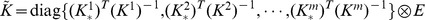, 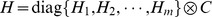, 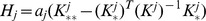, 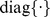 denotes block diagonal matrix.

Thus, the probability 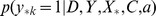 that protein 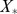 belongs to subcellular location 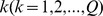 may be predicted by averaging out 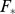, i.e.,
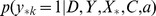


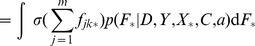
(13)

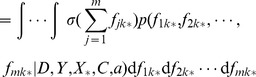
Notice that the predictive probability (??) also can not be computed analytically. In this paper, we resort to Monte Carlo sampling method to compute it.

Until now, we have presented the whole IMMMLGP algorithm under the assumption that *C*, *a* and 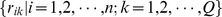 have been obtained. The details of computing *C*, *a* and 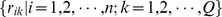 can be found in **Appendix S1**.

**Figure 2:**
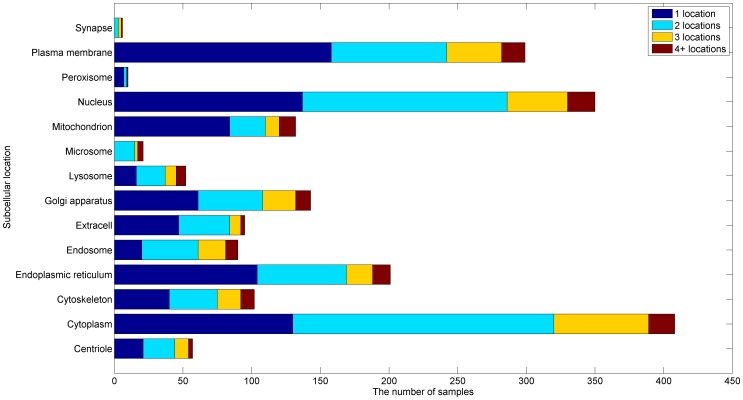
Subcellular distribution of the test samples.

**Table 2:** The performance comparison between the proposed algorithm and Hum-mPLoc 2.0.

Evaluation metric		The proposed algorithm	Hum-mPLoc 2.0
The whole test set	Average precision 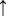	**0.581**	0.579
	Recall 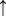	**0.643**	0.519
	F1-score 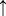	0.506	**0.541**
	Absolute true success rate 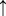	0.202	**0.294**
	Coverage 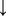	**4.303**	5.317
	Ranking loss 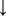	**0.419**	0.496
Samples with multiple sites	Average precision 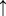	**0.596**	0.568
	Recall 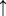	**0.579**	0.443
	F1-score 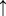	**0.576**	0.548
	Absolute true success rate 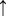	**0.153**	0.114
	Coverage 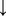	**6.800**	8.453
	Ranking loss 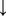	**0.463**	0.568

## Results and Discussion

In this section, we test the proposed algorithm on a human protein data set collected from the Swiss-Prot database by Shen and Chou [47]. This data set includes 3106 different protein sequences covering 14 subcellular locations, where 2580 proteins belong to one subcellular location, 480 to two locations, 43 to three locations, and 3 to four locations. None of proteins included here has >25% pairwise sequence identity to any other in a same subcellular location. Five feature extraction technologies including GO process, GO function, GO component, composition of amino acids, and pseudo amino acid composition with 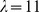, which measure the similarity of proteins from different aspects, are chosen in the experiments. The details of these feature extraction technologies can be found in [61] or [62]. In each experiment, the approach proposed in [63] is used to determine the parameter 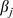 of covariance function 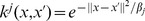.

In statistical prediction, the following three cross-validation methods are often used to examine a predictor for its effectiveness in practical application: independent data set test, subsampling test, and jackknife test [64]. Of the three test methods, the jackknife test is deemed the most objective [65]. The reasons are as follows. (i) For the independent data set test, although all the proteins used to test the predictor are outside the training data set used to train it so as to exclude the “memory” effect or bias, the way of how to select the independent proteins to test the predictor could be arbitrary unless the number of independent proteins is sufficiently large. This kind of arbitrariness might result in completely different conclusions. For instance, a predictor achieving a higher success rate than the other predictor for a given independent test data set might fail to keep so when tested by another independent test data set [64]. (ii) For the subsampling test, the concrete procedure usually used in literatures is the 2-fold, 5-fold, 7-fold or 10-fold cross-validation. The problem with this kind of subsampling test is that the number of possible selections in dividing a benchmark data set is an astronomical figure even for a very simple data set, as elucidated in [65] and demonstrated by Equations (28)-(30) in [59]. Therefore, in any actual subsampling cross-validation tests, only an extremely small fraction of the possible selections are taken into account. Since different selections will always lead to different results even for a same benchmark data set and a same predictor, the subsampling test cannot avoid the arbitrariness either. A test method unable to yield a unique outcome cannot be deemed as a good one. (iii) In the jackknife test, all the proteins in the benchmark data set will be singled out one-by-one and tested by the predictor trained by the remaining protein samples. During the process of jackknifing, both the training data set and test data set are actually open, and each protein sample will be in turn moved between the two. The jackknife test can exclude the “memory” effect. Also, the arbitrariness problem as mentioned above for the independent data set test and subsampling test can be avoided because the outcome obtained by the jackknife cross-validation is always unique for a given benchmark data set. Accordingly, the jackknife test has been increasingly and widely used by those investigators to examine the quality of various predictors (see, e.g., [4]–[10], [66]–[72]). However, to reduce the computational time, we will adopt the independent data set and subsampling test methods to examine the proposed predictor as done by many predictors with SVM or Bayesian network as the classifier [42], [50]. And we will try to prevent the influence of the arbitrariness problem mentioned above on the experimental results through constructing an independent data set as large as possible or repeating subsampling test many times.

**Table 3:** Some examples of the experimental results outputted by the two algorithms.

Accession number	Locations annotated in Swiss-Prot database	The predicted results ofHum-mPLoc 2.0	The predicted results of theproposed algorithm
P60852	Plasma membrane; Extracell	Extracell	Plasma membrane; Extracell
O75396	Endoplasmic reticulum; Golgi apparatus	Endoplasmic reticulum	Endoplasmic reticulum; Golgi apparatus
Q2VWA4	Cytoplasm; Nucleus	Nucleus	Cytoplasm; Nucleus
Q6NT55	Endoplasmic reticulum; Microsome	Endoplasmic reticulum; Microsome;Extracell	Endoplasmic reticulum; Microsome
P42261	Plasma membrane; Endoplasmic reticulum; Synapse	Plasma membrane; Synapse; Extracell	Plasma membrane; Endoplasmic reticulum; Synapse
Q9Y3A5	Cytoplasm; Nucleus; Cytoskeleton	Mitochondrion	Cytoplasm; Nucleus
P49419	Cytoplasm; Nucleus; Mitochondrion	Mitochondrion	Cytoplasm; Mitochondrion
Q86WV6	Endoplasmic reticulum; Cytoplasm; Mitochondrion; Plasma membrane	Cytoplasm	Cytoplasm; Endoplasmic reticulum
Q99527	Plasma membrane; Golgi apparatus; Endoplasmic reticulum	Plasma membrane	Plasma membrane; Endoplasmic reticulum
O75410	Cytoplasm; Nucleus; Centriole	Nucleus	Cytoplasm; Nucleus; Centriole; Mitochondrion

Since the performance evaluation of multi-label problems is much more complicated than the traditional single-label ones, the following popular multi-label evaluation metrics are used to comprehensively evaluate the performance of the proposed approach. Here, 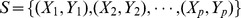 denotes a test set, 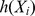 returns a set of proper labels of *X_i_*; 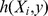 returns a probability indicating the confidence for *y* to be a proper label of *X_i_*; 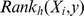 is the rank of *y* derived from 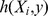.

- Average precision: 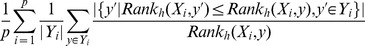. It can compute the average fraction of labels ranked above a particular label 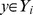.
- Coverage: 
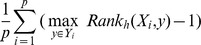
. It can evaluate how far one needs to go in the list of labels in order to cover all the proper labels of a sample.
- Ranking loss: 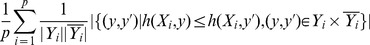, where 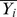 is the complementary set of Yi. It can evaluate the average fraction of label pairs that are not correctly ordered for a sample.
- Recall: 
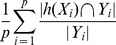
.
- F1-score: 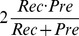, where 
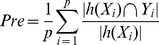
 and 
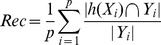
.
- Absolute true success rate: 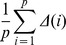, where 
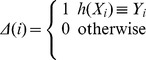
. According to the definition, the prediction score of a test protein can be counted as 1 when and only when all its subcellular locations are exactly predicted without any underprediction or overprediction. Therefore, the absolute true success rate is much more strict and harsh than other metrics.

The more detailed definitions of the first five metrics can be found in [73] and [74], and the definition of absolute true success rate can be found in [58] or [53].

As shown in the Section “Methods”, a main contribution of the proposed approach is that the correlations among the locations are exploited by using a covariance matrix *C*. In order to justify the fact that the superior performance of the proposed algorithm benefits by considering the correlations among labels, we firstly investigate the performance difference between the proposed approach and its variation in which the covariance matrix *C* is assumed to be an identity matrix (i.e., the locations are considered as mutually independent ones). Table 1 shows the experimental results on the human protein data set. For each evaluation metric, ‘
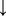
’ indicates ‘the smaller the better’ while ‘
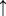
’ indicates ‘the bigger the better’. In our experiments, the data were randomly partitioned in half to form a training set and a test set. We repeated each experiment for 5 random splits, and reported the average of the results obtained over 5 different test sets. In order to study the influence of the percentage of the proteins with multiple sites on the proposed approach, we construct a new human protein data set which contains around 40% proteins with multiple sites by randomly removing some proteins with single site from the original data set. Table 1 also presents the experimental results on this new data set. It can be seen from Table 1 that the proposed approach can achieve superior performance than its variation no matter on the whole test set or the test samples with multiple sites only. Moreover, the performance gap tends to increase when the percentage of the proteins with multiple sites increases. Thus, as what we expected, the correlations among the locations are the useful information for improving the prediction accuracy of the predictor and the covariance matrix could exploit this information effectively.

In order to evaluate the relative performance of the proposed algorithm, it is compared with an existing algorithm named Hum-mPLoc 2.0 [47], which is a popular web-server predictor for the subcellular localization prediction of human proteins with multiple sites. Since the whole human data set has been taken as the training set of Hum-mPLoc 2.0, to make a fair and comprehensive comparison, we have to take it as the training set of the proposed algorithm also and construct a test set according to the following criteria: (1) they must belong to human proteins, as clearly annotated in Swiss-Prot database; (2) None of proteins included here has >25% sequence identity to the ones of the training set in a same subcellular location. By following the above procedures, we obtained a test set containing 1315 proteins, of which 825 located to one site, 369 to two sites, 91 to three sites, and 30 to more than three sites. The details about the distribution of these samples can be seen in Fig. 2. Table 2 presents the experimental results of the proposed algorithm and Hum-mPLoc 2.0, where the best result on each metric is shown in bold face. It can be seen from Table 2 that the proposed algorithm achieves the best performance on four of the six evaluation metrics as far as the whole test set is concerned. Since these evaluation metrics measure the performance of algorithms from different aspects, one algorithm usually is difficult to outperform another on all the metrics. Thus, overall, the proposed algorithm can achieve superior performance than Hum-mPLoc 2.0 on this test set. In addition, Table 2 also presents the experimental results of each algorithm on the test samples with multiple sites only. It can be seen that the proposed algorithm consistently outperforms Hum-mPLoc 2.0 on the samples with multiple sites in terms of all evaluation metrics. This suggests that the proposed algorithm has the obvious advantage than Hum-mPLoc 2.0 for predicting the subcellular locations of proteins with multiple sites.

In order to understand why the proposed algorithm can achieve superior performance than Hum-mPLoc 2.0 on the proteins with multiple sites, we analysis the difference of the results outputted by the two algorithm. Table 3 shows some examples of the experimental results outputted by them. For the first 5 proteins, all their sites are correctly identified by the proposed algorithm but only partial sites can be correctly predicted by Hum-mPLoc 2.0. For the others, all of the two algorithms only can correctly predict their partial sites or incorrectly predict all their sites. It can be seen from Table 3 that the proposed algorithm can output as much as possible corrected locations than Hum-mPLoc 2.0 in most cases. For example, according to the experimental annotation in Swiss-Prot, the protein with accession number P60852 belongs to two locations: Plasma membrane and Extracell. If using Hum-mPLoc 2.0 to predict its sites, the output is ‘Extracell’, and ‘Plasma membrane’ is missed; however, the proposed algorithm can correctly output all of them. This may be the main reason why the proposed algorithm achieves superior performance than Hum-mPLoc 2.0.

Finally, it should be pointed out that although the proposed algorithm can achieve superior performance than the existing ones, it mainly benefits by the novel classifier but not the feature information. In the future, we will try to improve the algorithm by using more feature information such as FunD (functional domain) representation and SeqEvo (sequential evolution) representation. Moreover, since user-friendly and publicly accessible web-servers represent the future direction for developing practically more useful models, simulated methods, or predictors [75], we shall make efforts in our future work to provide a web-server for the method presented in this paper.

## Supporting Information

Appendix S1 (PDF)
