# Supplementary material for: Imbalanced Multi-Modal Multi-Label Learning for Subcellular Localization Prediction of Human Proteins with Both Single and Multiple Sites
Source: PLoS One. 2012 Jun 8;7(6):e37155. doi: 10.1371/journal.pone.0037155 (PMC3371015; doi:10.1371/journal.pone.0037155)
Supplement: Appendix S1 — (PDF) [file pone.0037155.s001.pdf]

Appendices

Solving $\hat{F}$ and A

Since the marginal likelihood $p(Y|D,C,a)$ is independent of F, we only need to consider the un-normalized posterior when maximizing posterior distribution (9) with regard to F, i.e., $\hat{F}$ may be obtained by maximizing $\psi(F)$

$$\begin{aligned}\psi(F) &\triangleq \log p(Y|F) + \log p(F|D,C,a)\\ &= \log p(Y|F) - \tfrac{1}{2}\sum_{j=1}^{m}(F_j^T((a_jK^j)\otimes C)^{-1}F_j + \log|(a_jK^j)\otimes C| + nQ\log 2\pi)\end{aligned} \tag{14}$$

By differentiating (14) with regard to F,

$$\nabla\psi(F) = \nabla\log p(Y|F) - (\mathrm{diag}\{a_1K^1, a_2K^2, \cdots, a_mK^m\}\otimes C)^{-1}F \tag{15}$$

$$\nabla\nabla\psi(F) = \nabla\nabla\log p(Y|F) - (\mathrm{diag}\{a_1K^1, a_2K^2, \cdots, a_mK^m\}\otimes C)^{-1} \tag{16}$$

where, $\nabla\log p(Y|F) = \mathbf{1}\otimes d$, $\mathbf{1}$ is an m dimensional column vector of ones, $d = [d_{11}, d_{12}, \cdots, d_{1Q}, d_{21}, \cdots, d_{2Q}, \cdots, d_{n1}, \cdots, d_{nQ}]^T$, $W \triangleq -\nabla\nabla\log p(Y|F) = (\mathbf{1}\cdot\mathbf{1}^T)\otimes W_0$, $W_0 = \mathrm{diag}\{w_{11}, w_{12}, \cdots, w_{1Q}, w_{21}, \cdots, w_{2Q}, \cdots, w_{n1}, \cdots, w_{nQ}\}$, $d_{ik} = r_{ik}y_{ik}/(1+\exp(y_{ik}\sum_{j=1}^{m} f_{jki}))$, $\exp(\cdot)$ denotes exponential function, $w_{ik} = r_{ik}\exp(y_{ik}\sum_{j=1}^{m} f_{jki})/(1+\exp(y_{ik}\sum_{j=1}^{m} f_{jki}))^2$. Thus, $\hat{F}$ is the solution of equation $\nabla\psi(F) = 0$, i.e.,

$$\hat{F} = (\mathrm{diag}\{a_1K^1, a_2K^2, \cdots, a_mK^m\}\otimes C)\nabla\log p(Y|\hat{F}) \tag{17}$$

we can use the Newton's method to solve equation (17), with the iteration

$$F_{new} = F - \lambda(\nabla\nabla\psi)^{-1}\nabla\psi = (1-\lambda)F + \lambda\begin{pmatrix} a_1(K^1\otimes C)h \\ a_2(K^2\otimes C)h \\ \vdots \\ a_m(K^m\otimes C)h \end{pmatrix} \tag{18}$$

where, $h = d + W_0(\sum_{j=1}^{m} F_j) - W_0^{\frac{1}{2}}B^{-1}W_0^{\frac{1}{2}}((\sum_{j=1}^{m} a_jK^j)\otimes C)(d + W_0(\sum_{j=1}^{m} F_j))$, $B = E + W_0^{\frac{1}{2}}((\sum_{j=1}^{m} a_jK^j)\otimes C)W_0^{\frac{1}{2}}$, λ is the step length. λ can be obtained by maximizing

$$\varphi(\lambda) \triangleq \psi(F - \lambda(\nabla\nabla\psi(F))^{-1}\nabla\psi(F)) \tag{19}$$

Here, the dichotomy method is used to solve λ.

By substituting $\hat{F}$ into the negative Hessian matrix(16), the matrix A may be obtained

$$A = (W + (\mathrm{diag}\{a_1K^1, a_2K^2, \cdots, a_mK^m\}\otimes C)^{-1})|_{F=\hat{F}} \tag{20}$$

Learning matrix C and coefficients a

In GP model, the hyperparameters usually can be obtained by maximizing the marginal likelihood (10). Since the integral in marginal likelihood is intractable, one way to achieve this is to provide a lower bound for the marginal likelihood and then solve parameters by maximizing the lower bound. In this paper, the lower bound Z obtained by Kim et al. [Kim HC, Ghahramani Z (2006) Bayesian Gaussian

process classification with the EM-EP algorithm. IEEE Transactions on Pattern Analysis and machine Intelligence 28: 1948-1959] will be used, i.e.,

$$\begin{aligned}
&\log p(Y|D,C,a)\\
&= \log \int p(Y|F)p(F|D,C,a)\mathrm{d}F\\
&= \log \int q(F|D,Y,C,a)\frac{p(Y|F)p(F|D,C,a)}{q(F|D,Y,C,a)}\mathrm{d}F\\
&\geq \int q(F|D,Y,C,a)\log\frac{p(Y|F)p(F|D,C,a)}{q(F|D,Y,C,a)}\mathrm{d}F \qquad (21)\\
&= \int q(F|D,Y,C,a)\log p(Y|F)\mathrm{d}F + \int q(F|D,Y,C,a)\log p(F|D,C,a)\mathrm{d}F\\
&\quad \cdot - \int q(F|D,Y,C,a)\log q(F|D,Y,C,a)\mathrm{d}F\\
&=: \log Z
\end{aligned}$$

In addition, because the parameters $\hat{F}$ and A of $q(F|D,Y,C,a)$ are also the functions of C and a, it is difficult to maximize $\log Z$ directly. Here, an EM-like algorithm is used to solve this problem. In the E-step, we compute the values of $\hat{F}$ and A by using (18) and (20) given the parameters C and a. In the M-step, C and a are updated by maximizing the lower bound $\log Z$ where $\hat{F}$ and A is respectively fixed as the values of $\hat{F}$ and A obtained in the E-step. The E-step and M-step are alternated until convergence. Since the terms $\int q(F|D,Y,C,a)\log p(Y|F)\mathrm{d}F$ and $\int q(F|D,Y,C,a)\log q(F|D,Y,C,a)\mathrm{d}F$ in (21) are only related to $\hat{F}$ and A, we only need to maximize $\int q(F|D,Y,C,a)\log p(F|D,C,a)\mathrm{d}F$ in the M-step. By substituting (2) and (11) into it, we obtain

$$\begin{aligned}
&\textstyle\int q(F|D,Y,C,a)\log p(F|D,C,a)\mathrm{d}F\\
&= -\tfrac{1}{2}\sum_{j=1}^{m}(\log|2\pi a_j K^j \otimes C| + \hat{F}_j^T(a_j K^j\otimes C)^{-1}\hat{F}_j + \mathrm{tr}(\bar{A}_{jj}(a_jK^j\otimes C)^{-1}))\\
&= -\tfrac{1}{2}\sum_{j=1}^{m}(\log|2\pi a_j K^j \otimes C| + \mathrm{tr}(E\otimes(a_j^oC^o(a_jC)^{-1})) - \mathrm{tr}(G(K^j\otimes((a_j^o)^2C^o(a_jC)^{-1}C^o)))) \qquad (22)\\
&= -\tfrac{1}{2}\sum_{j=1}^{m}(nQ\log(2\pi) + Q\log|K^j| + n\log|C| + nQ\log a_j + n\mathrm{tr}(R_j(a_jC)^{-1}))\\
&=: \bar{Z}(C,a|\hat{F},A)
\end{aligned}$$

where, C^o and a_j^o respectively denote the values of C and a_j obtained in the last M step; $\{\bar{A}_{ls}|l,s = 1,2,\cdots,m\}$ are square matrixes of order nQ by which A^{-1} is expressed with block, i.e., $A^{-1} = (\bar{A}_{ls})_{m\times m}$; $G = \hat{W}_0^{\frac{1}{2}}\hat{B}^{-1}\hat{W}_0^{\frac{1}{2}} - \hat{d}\hat{d}^T$, $\hat{d} = d|_{\hat{F}}$, $\hat{W}_0 = W_0|_{\hat{F}}$, $\hat{B} = E + \hat{W}_0^{\frac{1}{2}}(\sum_{j=1}^{m} a_jK^j)\otimes C^o\hat{W}_0^{\frac{1}{2}}$; $R_j = a_j^oC^o - \frac{1}{n}(a_j^o)^2C^o(\sum_{l=1}^{n}\sum_{s=1}^{n}K_{ls}^jG_{ls})C^o$, $\{G_{ls}\}$ are square matrixes of order Q by which G is expressed with block.

By differentiating $\bar{Z}(C,a|\hat{F},A)$ with regard to C, we have

$$\frac{\partial\bar{Z}(C,a|\hat{F},A)}{\partial C} = -\frac{n}{2}\sum_{j=1}^{m}(C^{-1} - \frac{1}{a_j}C^{-1}R_jC^{-1}), \qquad (23)$$

Thus, we can obtain

$$C = \frac{1}{m}\sum_{j=1}^{m}a_j^{-1}R_j \qquad (24)$$

at the maximum of $\bar{Z}(C, a|\hat{F}, A)$.

By substituting (24) into (22), we have

$$\bar{\bar{Z}}(a|\hat{F}, A) \triangleq -\frac{1}{2}\sum_{j=1}^{m}(nQ\log(2\pi) + Q\log|K^j| + n\log|\frac{1}{m}\sum_{j=1}^{m}a_j^{-1}R_j| + nQ\log a_j + nmQ) \quad (25)$$

So, we can obtain a by maximizing $\bar{\bar{Z}}(a|\hat{F}, A)$ firstly, and then obtain C based on the equation (24). In this paper, the conjugate gradient method was used to solve a. In addition, in order to find a unique solution, a_1 was set to 1.

Computing r_{ik}

It can be seen from the above Section that the posterior mean $\hat{F}$ can be obtained by minimizing the function

$$\bar{\psi}(F) \triangleq \frac{1}{2}\sum_{j=1}^{m}F_j^T(a_jK^j \otimes C)^{-1}F_j - \sum_{i=1}^{n}\sum_{k=1}^{Q}\log p(y_{ik}|f_{1ki}, f_{2ki}, \cdots, f_{mki}) \quad (26)$$

In the view of regularization, the first term of (26) represents smoothness assumption on F as encoded by a suitable reproducing kernel Hilbert space, and the second term is a data-fit term assessing the quality of the prediction F for the observed data Y. In the case of imbalanced data, the posterior mean $\hat{F}$ obtained by using (26) will tend to be overwhelmed by the majority classes. In order to deal with this problem, an intuitional idea is to make errors of fitting on minority-class data costlier than errors on majority-class data by setting different weighting coefficient for the data of different classes, i.e., change the second term to $\sum_{i=1}^{n}\sum_{k=1}^{Q} r_{ik}\log p(y_{ik}|f_{1ki}, f_{2ki}, \cdots, f_{mki})$. If we notice the fact that $\sum_{i=1}^{n}\sum_{k=1}^{Q}\log p(y_{ik}|f_{1ki}, f_{2ki}, \cdots, f_{mki})$ is the logarithm of likelihood (5), the reason for dealing with the imbalance of data by using the likelihood (6) will be clear.

In this paper, r_{ik} is computed as follows

$$r_{ik} = \begin{cases} n_i^-/n_i^+ & y_{ik} = 1 \\ 1 & y_{ik} = -1 \end{cases} \quad (27)$$

where $n_i^+ = |\{y_{ik}|y_{ik} = 1, k = 1, 2, \cdots, n\}|$ and $n_i^- = |\{y_{ik}|y_{ik} = -1, k = 1, 2, \cdots, n\}|$ denote the numbers of positive samples and negative samples for the ith location, respectively; $|\cdot|$ denotes the cardinality of a set.

Reducing the computational complexity of IMMMLGP

Because of the need to invert a $Qn \times Qn$ matrix B, the computational complexity of training the IMMMLGP algorithm is about $O((Qn)^3)$ which is prohibited by the problems with large data set. For the problem with moderate n, we can reduce the computational complexity of inverting the $Qn \times Qn$ matrix B by approximating $\sum_{j=1}^{m}a_jK^j$ in the form $\sum_{j=1}^{m}a_jK^j \approx PP^{\mathrm{T}}$, here, P is an $n \times n_0$ matrix, $n_0 \ll n$. Notice that, by representing $(\sum_{j=1}^{m}a_jK^j)$ with P, B^{-1} can be expressed as

$$B^{-1} = E - W_0^{\frac{1}{2}}(P \otimes L)B_1^{-1}(P^{\mathrm{T}} \otimes L^{\mathrm{T}})W_0^{\frac{1}{2}} \quad (28)$$

where, $B_1 = I + (P^{\mathrm{T}} \otimes L^{\mathrm{T}})W_0(P \otimes L), C = LL^{\mathrm{T}}$. Thus, the problem is transformed into the inversion of a $Qn_0 \times Qn_0$ matrix. In this paper, the optimal reduced-rank approximation $U_{n_0}\Lambda_{n_0}U_{n_0}^{\mathrm{T}}$ of $\sum_{j=1}^{m}a_jK^j$

with respect to the Frobenius norm is used to obtain P, where Λ_{n_0} is the diagonal matrix of the leading n_0 eigenvalues of $\sum_{j=1}^{m} a_j K^j$ and U_{n_0} is the matrix of the corresponding eigenvectors. Thus, $P = U_{n_0}\Lambda_{n_0}^{1/2}$. Unfortunately, this is limited for the problem with large n because the computation of the eigendecomposition is a $O(n^3)$ operation. The Bayesian committee machine (BCM)[Tresp V (2000) A bayesian committee machine. Neural Computation 12: 2719-2741] can be used to improve the IMMMLGP model for dealing with the problems with large n. Instead of training one classifier using the whole training data set, the idea of BCM is to split up the training data set into several data subsets and train one sub-classifier on each of them, then combine the predictions of these individual sub-classifiers using a weighting scheme.

Let $\{S_1, S_2, \cdots, S_L\}$ be a partition of the training data set S. C_α and a_α respectively denote the values of C and a obtained on the training subset $\bigcup_{l\in\alpha} S_l$, $\alpha \subset \{1, 2, \cdots, L\}$. Based on the Bayesian committee machine, we can obtain the following approximation of the distribution (12)

$$p(F_*|S, X_*, C, a) = p(F_*|\bigcup_{l=1}^{L} S_l, X_*, C_{\{1,\cdots,L\}}, a_{\{1,\cdots,L\}}) \approx const \times \frac{\prod_{l=1}^{L} p(F_*|S_l, X_*, C_{\{l\}}, a_{\{l\}})}{\prod_{l=2}^{L} p(F_*|X_*, C_{\{l\}}, a_{\{l\}})} = \mathcal{N}(F_*|\mu, \sigma) \tag{29}$$

where, $const$ is a constant, $\mu = \sigma(\sum_{l=1}^{L} \mathbb{V}[F_*|S_l, X_*, C_{\{l\}}, a_{\{l\}}]^{-1}\mathbb{E}[F_*|S_l, X_*, C_{\{l\}}, a_{\{l\}}])$, $\mathbb{E}[\cdot]$ and $\mathbb{V}[\cdot]$ denote the mean and covariance respectively, $\sigma = (-\sum_{l=2}^{L} \mathbb{V}[F_*|X_*, C_{\{l\}}, a_{\{l\}}]^{-1} + \sum_{l=1}^{L} \mathbb{V}[F_*|S_l, X_*, C_{\{l\}}, a_{\{l\}}]^{-1})^{-1}$.

Thus, for the problems with large training set, we can compute the predictive probability (13) by using (29) instead of (12).
